# Targeting mTOR with MLN0128 Overcomes Rapamycin and Chemoresistant Primary Effusion Lymphoma

**DOI:** 10.1128/mBio.02871-18

**Published:** 2019-02-19

**Authors:** Carolina Caro-Vegas, Aubrey Bailey, Rachele Bigi, Blossom Damania, Dirk P. Dittmer

**Affiliations:** a UNC Lineberger Comprehensive Cancer Center, University of North Carolina, Chapel Hill, North Carolina, USA; b Department of Microbiology and Immunology, University of North Carolina, Chapel Hill, North Carolina, USA; Princeton University; University of Virginia; University of Southern California

**Keywords:** INK128, Kaposi's sarcoma-associated herpesvirus, MLN0128, sapanisertib, everolimus, lymphoma, mTOR, rapamycin, sirolimus

## Abstract

Primary effusion lymphoma (PEL) is caused by Kaposi’s sarcoma-associated herpesvirus (KSHV). PEL has a highly active mTOR pathway, which makes mTOR a potential therapeutic target. MLN0128 is an ATP-competitive inhibitor of mTOR that has entered clinical trials for solid tumors. Our results demonstrated that MLN0128 has a greater effect on inhibiting proliferation than the allosteric mTOR inhibitor rapamycin. MLN0128 has ∼30 nM 50% inhibitory concentration (IC_50_) across several PEL cell lines, including PEL that is resistant to conventional chemotherapy. MLN0128 induced apoptosis in PEL, whereas rapamycin induced G_1_ arrest, consistent with a different mechanism of action. MLN0128 inhibited phosphorylation of mTOR complex 1 and 2 targets, while rapamycin only partially inhibited mTOR complex 1 targets. PEL xenograft mouse models treated with MLN0128 showed reduced effusion volumes in comparison to the vehicle-treated group. Rapamycin-resistant (RR) clones with an IC_50_ for rapamycin 10 times higher than the parental IC_50_ emerged consistently after rapamycin exposure as a result of transcriptional adaptation. MLN0128 was nevertheless capable of inducing apoptosis in these RR clones. Our results suggest that MLN0128 might offer a new approach to the treatment of chemotherapy-resistant PEL.

## Abstract (precis)

Primary effusion lymphoma (PEL) is an aggressive and incurable malignancy, which is usually characterized by lymphomatous effusions in body cavities without tumor masses. PEL has no established treatment and a poor prognosis, with a median survival time shorter than 6 months. PEL usually develops in the context of immunosuppression, such as HIV infection or post-organ transplantation. The optimal treatment for PEL has not been established, as PEL is generally resistant to traditional chemotherapy. The molecular drivers for PEL are still unknown; however, PEL displays a constitutively active mammalian target of rapamycin (mTOR) pathway, which is critical for metabolic and cell survival mechanisms. Therefore, the evaluation of novel agents targeting the mTOR pathway could be clinically relevant for the treatment of PEL.

## INTRODUCTION

Primary effusion lymphoma (PEL) is a non-Hodgkin B-cell lymphoma that is associated with infection by Kaposi sarcoma-associated herpesvirus (KSHV) (1). PEL typically presents as lymphomatous effusions in body cavities, most commonly in the pleural, pericardial, and peritoneal cavities. This lymphoma generally develops in the context of immunosuppression, such as during HIV infection or organ transplantation (2). Currently, PEL is treated with a chemotherapeutic regimen containing cyclophosphamide, doxorubicin, vincristine, and prednisolone (CHOP). Resistance develops rapidly, with a median survival time of 6.2 months (3, 4). Hence, there exists an urgent need to develop novel and targeted therapies for PEL.

PEL are addicted to the mammalian target of rapamycin (mTOR) signaling pathway. All PEL tested display constitutively active mTOR (5, 6). The mTOR pathway controls cell growth, metabolism, proliferation, angiogenesis, and survival (reviewed in reference 7) and is activated in a large number of cancers. In nonviral cancers, this is often due to activating mutations upstream of mTOR and/or loss of tumor suppressors such as the phosphatase and tensin homolog (PTEN). In PEL, PTEN is intact but inactivated by phosphorylation (8). Several viral proteins, such as ORFK1 (K1) (9), ORFK2 (vIL6) (9), ORF45 (10), and ORF74 (vGPCR) (11–13), induce the mTOR pathway in PEL as well as in Kaposi’s sarcoma (KS).

The mTOR kinase functions in two structurally and functionally distinct complexes designated mTOR complexes 1 (mTORC1) (14, 15) and 2 (mTORC2) (16, 17). mTORC1 targets p70-S6 kinase 1 (p70S6K1) and eukaryotic translation initiation factor 4E-binding protein 1 (4EBP1). mTORC1 phosphorylates p70S6K1 at Thr389, which in turn phosphorylates ribosomal protein S6 (RPS6), thereby promoting protein synthesis (18). mTORC1 also phosphorylates 4EBP1 at multiple sites, which causes dissociation from eukaryotic translation initiation factor 4E (eIF4E), promoting cap-dependent translation. mTORC2 phosphorylates Akt (also known as protein kinase B) at Ser473. Akt in turn activates mTORC1 through a positive-feedback loop (19, 20), as well as additional prosurvival proteins. Another mTORC2 target is SGK1. AKT and SGK1 are members of the AGC family of kinases. They have both distinct and overlapping roles (21, 22). mTORC2 phosphorylates SGK1 proteins at Ser422, as well as one of SGK1’s physiological targets, N-myc downstream-regulated gene 1 (NDRG1) (23). Phosphorylation of these proteins represents the best understood biomarker of mTORC activity.

Rapamycin and its analogs (rapalogs) are allosteric inhibitors of mTORC1. We and others previously reported that rapamycin had preclinical activity against in PEL, as well as clinical efficacy against KS (5, 12, 24–26). Switching from cyclosporine to rapamycin has become the standard of care for transplant-associated KS. Unfortunately, rapamycin only arrests cells: it does not induce apoptosis, and resistance develops readily (27). While rapamycin always inhibits p70S6K1 and RPS6, its effect on 4EBP1 is more variable (28–30). Since rapamycin does not target mTORC2, this may result in a compensatory activation of Akt and mTORC2, at least until enough mTOR kinase is trapped in inactive rapamycin-FKBP-mTORC1 complexes to start depleting mTORC2 (31). These limitations triggered the development of second-generation, ATP-competitive inhibitors, which target both mTORC1 and mTORC2.

This is the study of an ATP-competitive inhibitor in PEL. We focused on MLN0128 (INK128 [sapanisertib]), rather than other ATP-competitive inhibitors, because MLN0128 is orally bioavailable and farthest along in clinical development (32–34). MLN0128 inhibited both mTORC1 and mTORC2 in PEL at a nanomolar 50% inhibitory concentration (IC_50_) and induced rapid apoptosis rather than G_1_ arrest. MLN0128 was efficacious against doxorubicin-resistant PEL, such as the BCP-1 isolate, as well as rapamycin-resistant PEL. Furthermore, MLN0128 had reproducible efficacy in PEL xenograft models. Based on these studies, the clinical evaluation of MLN0128 in PEL and KS seems warranted.

## RESULTS

### MLN0128 inhibits PEL proliferation.

**FIG 1:**
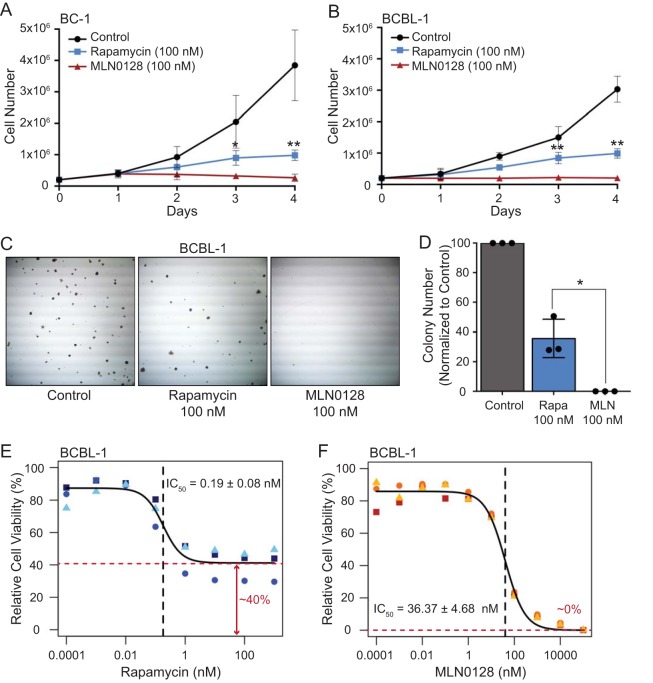
MLN0128 inhibits PEL proliferation. (A) BC-1 and (B to D) BCLB-1 cells were incubated with 100 nM rapamycin or MLN0128, and inhibition of cell proliferation was assessed by (A and B) trypan blue exclusion assay at the indicated times or (C and D) colony formation assay after 2 weeks. Data represent the mean ± standard deviation (SD) from *n* = 3 independent experiments (unpaired 2-tailed *t* test; *, *P* < 0.05, and **, *P* < 0.01, rapamycin versus MLN0128 group). BCBL-1 cells were treated with increasing concentrations of (E) rapamycin or (F) MLN0128 for 48 h, and cell viability was measured by CellTiter-Glo luminescent cell viability assay. Dose-response curves were generated as a percentage of the vehicle (100%) and no-cell control (0%) in R. Each data point represents the mean from independent wells (*n* = 4).

**FIG 2:**
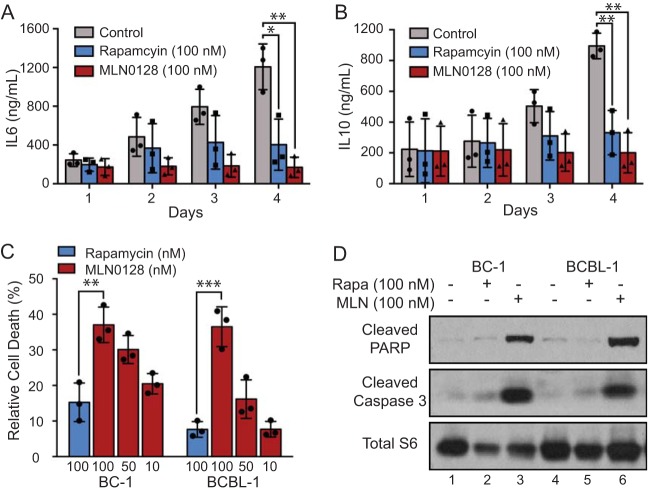
MLN0128 induces PEL apoptosis. (A and B) BC-1 cells were treated with 100 nM rapamycin or MLN0128 for the indicated times, (A) IL-6 and (B) IL-10 (PEL markers) on the supernatant were measured by ELISA. Data represent the mean ± SD from *n* = 3 independent experiments (unpaired 2-tailed *t* test; *, *P* < 0.05, **, *P* < 0.01, and ***, *P* < 0.001, control versus rapamycin or MLN0128 group). BC-1 and BCBL-1 cells were treated with the indicated concentration of rapamycin and MLN0128 for 48 h; cell apoptosis was measured by (C) annexin V fluorescence-activated cell sorter (FACS), where percentages indicate annexin V-positive cells (apoptotic) normalized to control, and (D) expression of cleaved PARP, cleaved caspase-3, and total S6 as the loading control. Data represent the mean ± SD from *n* = 3 independent experiments (unpaired 2-tailed *t* test; *, *P* < 0.05, **, *P* < 0.01, and ***, *P* < 0.001, rapamycin versus MLN0128 group). Western blots were repeated three times with similar results.

To compare MLN0128 and rapamycin, we tested several well-characterized PEL cell lines, such as BC-1 (KSHV and Epstein-Barr virus [EBV] positive) and BCBL-1 (KSHV positive, but EBV negative), across multiple experimental designs. (i) At 100 nM, MLN0128 inhibited PEL proliferation over time, as measured by trypan blue exclusion assay (Fig. 1A and B). (ii) MLN0128 abolished colony formation in soft agar. In contrast, rapamycin only reduced colony number to ∼40% of the control for BCBL-1 (Fig. 1C and D) and ∼20% of the control for BC-1 at the same concentration (see Fig. S1A and S1B in the supplemental material). (iii) CellTiter-Glo assay (CTG) confirmed the previously reported IC_50_ values for rapamycin (Fig. 1E; see Fig. S1C in the supplemental material). Of note, even at the highest concentration of rapamycin, there remained a resistant fraction of live cells, consistent with cell cycle arrest (see Fig. S2 in the supplemental material). MLN0128 had low nanomolar concentration IC_50_ values in this assay ranging from 9.71 to 47.56 nM (Table 1). In contrast to rapamycin, no resistant fraction remained in MLN0128-treated cells (Fig. 1F; see Fig. S1D in the supplemental material). Compared to two other rapalogs, temsirolimus and ridaforolimus, with improved solubility, MLN0128 still showed a more complete inhibition of PEL growth (Fig. S1E and S1F). All experiments were conducted at 0.00002% of dimethyl sulfoxide (DMSO), which up to 0.1% final concentration had no effect on cell growth (data not shown). (iv) Levels of interleukin-6 (IL-6) and IL-10 were evaluated as these cytokines correlate with PEL proliferation and may function as autocrine growth factors (35, 36). As reported previously, IL-6 and IL-10 were significantly reduced upon treatment with rapamycin. Treatment with MLN0128 showed even greater reduction (Fig. 2A and B). Importantly, when the data are normalized to the number of cells in each sample, the difference between IL-6 and IL-10 levels is not significant, which confirms IL-6 and IL-10 as markers for PEL proliferation. Whether in addition IL-10 is more directly sensitive to mTORC status remains the subject of further studies. These results demonstrate that MLN0128 inhibits PEL proliferation in a manner on par with or better than rapamycin, temsirolimus, and ridaforolimus.

**TABLE 1:** MLN0128 IC_50_ values for a panel of 5 PEL cell lines

PEL cell line (*n* = 3)	IC_50_ (nM)
BC-1	16.20 ± 4.94
BCBL-1	37.43 ± 13.13
BC-3	47.56 ± 7.48
BCP-1	9.71 ± 0.76
BCBL-1TrexRTA-Luc	26.07 ± 5.15

10.1128/mBio.02871-18.1 FIG S1 (A and B) BC-1 cells were incubated with 100 nM rapamycin or MLN0128, and inhibition of cell proliferation was assessed by colony formation assay after 2 weeks. Data represent the mean ± SD from *n* = 3 independent experiments (Student’s *t* test, *, *P* < 0.05, rapamycin versus MLN0128 group). BC-1 cells were treated with increasing concentrations of (C) rapamycin or (D) MLN0128 for 48 h, and cell viability was measured by the CellTiter-Glo luminescent cell viability assay. (E) BC-1 and (F) BCBL-1 cells were treated with increasing concentrations of MLN0128 (MLN), rapamycin (rapa), everolimus (evero), temsirolimus (tem), ridaforolimus (rida), and tacrolimus (tacro) for 48 h, and cell viability was measured by the CellTiter-Glo luminescent cell viability assay. Dose-response curves were generated as a percentage of the no-drug control (100%) and no-cell control (0%) in R. Data represent the mean from *n* = 4 independent wells. Download FIG S1, DOCX file, 0.7 MB. Copyright © 2019 Caro-Vegas et al. 2019 Caro-Vegas et al. This content is distributed under the terms of the Creative Commons Attribution 4.0 International license.

10.1128/mBio.02871-18.2 FIG S2 (A) BC-1 and (B) BCBL-1 cells were treated with 100 nM rapamycin and MLN0128 for 48 h. Cells were stained with propidium iodide and analyzed by FACS and FlowJo with the tool of cell cycle distribution. Representative FACS analysis in FlowJo for cell cycle distribution for BC-1 (C to E) and BCBL-1 (F to H) plots are live gated. Data represent the mean ± SD from *n* = 3 independent experiments (Student’s *t* test; *, *P* < 0.05, control versus MLN0128 group). Download FIG S2, DOCX file, 0.2 MB. Copyright © 2019 Caro-Vegas et al. 2019 Caro-Vegas et al. This content is distributed under the terms of the Creative Commons Attribution 4.0 International license.

### MLN0128, but not rapamycin, induces apoptosis of PEL cell lines.

To test the hypothesis that MLN0128 induced cell death, (i) we used annexin V/propidium iodide (PI) flow cytometry to quantify apoptosis at 48 h. MLN0128 induced significantly higher levels of annexin V-positive cells than rapamycin in a dose-dependent manner (Fig. 2C). Representative flow cytometric plots are shown in Fig. S3 in the supplemental material. (ii) Apoptosis was confirmed by Western blotting for cleaved poly(ADP-ribose) polymerase (PARP) and cleaved caspase-3, which are established markers of apoptosis (Fig. 2D). MLN0128 induced accumulation of cleaved PARP and cleaved caspase-3, while rapamycin did not. In sum, MLN0128 induced apoptosis, whereas rapamycin did not.

10.1128/mBio.02871-18.3 FIG S3 Gating strategy for delineating viable, early apoptotic, late apoptotic, and necrotic cells. Shown are representative FACS analysis results in FlowJo from the annexin V/PI assay. (A to C) BC-1 and (D to F) BCBL-1 cells were treated with 100 nM rapamycin or MLN0128 as indicated and incubated for 48 h. In each panel, the lower left quadrant (Q4) indicates viable cells (annexin negative, PI negative), the lower right quadrant (Q3) indicates early apoptotic cells (annexin positive, PI negative), the upper right quadrant (Q2) indicates late apoptotic cells (annexin positive, PI positive), and the upper left quadrant (Q1) indicates necrotic cells (annexin negative, PI positive). Download FIG S3, DOCX file, 0.3 MB. Copyright © 2019 Caro-Vegas et al. 2019 Caro-Vegas et al. This content is distributed under the terms of the Creative Commons Attribution 4.0 International license.

### MLN0128 inhibits mTORC1 and mTORC2 signaling.

**FIG 3:**
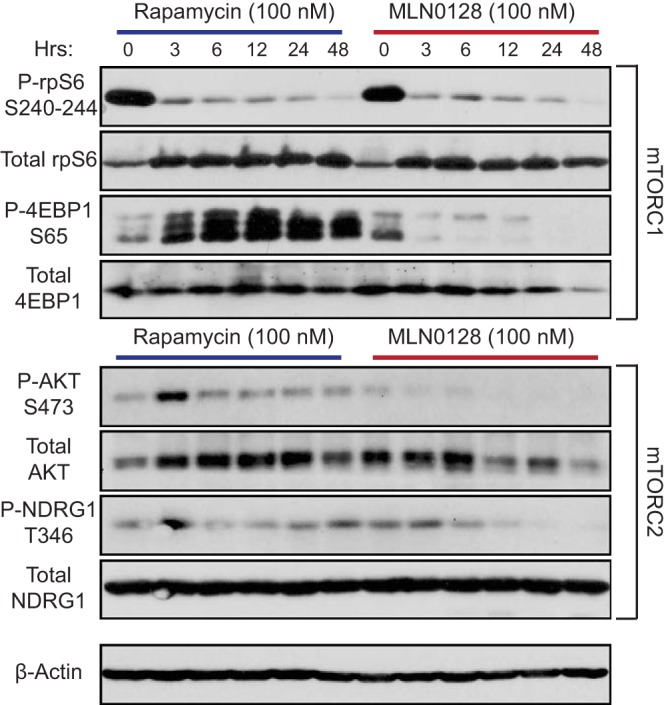
MLN0128 blocks both mTORC1 and mTORC2 in PEL. BCBL-1 cells were treated with 100 nM rapamycin or MLN0128 for 0 to 48 h, and regular and phospho levels of S6, 4EBP1, AKT, and NDGR1 were measured by Western blotting. β-Actin was used as a loading control for whole-cell extracts. Western blots were repeated three times with similar results.

To examine if MLN0128 inhibited mTORC1 and mTORC2 in PEL cells, phosphorylated and total levels of validated downstream targets of mTORC1 (rpS6 and 4EBP1) or mTORC2 (AKT and NGRD1) were measured. MLN0128 inhibited the phosphorylation of both mTORC1 targets, 4EBP1 (Ser65) and rpS6 (Ser240–244), as well as both mTORC2 targets, AKT (Ser473) and NDRG1 (Thr346) (Fig. 3). Rapamycin only partially inhibited mTORC1: phosphorylation of rpS6 was inhibited, yet 4EBP1 remained phosphorylated. As expected, rapamycin did not inhibit mTORC2. MLN0128 prevented feedback activation of AKT, while rapamycin led to the transient phosphorylation of AKT at Ser473 at 3 h. To confirm the specificity, we subjected MLN0128 to a high-throughput assay against 442 purified kinases (see Table S1 in the supplemental material) (37). MLN0128 was specific for mTOR at 25 nM, which was similar to the IC_50_ for PEL (see Fig. S4A in the supplemental material) (38). At 10,000 nM, MLN0128 showed a somewhat broader inhibition profile, similar to other mTOR/phosphatidylinositol 3-kinase (PI3K) ATP-competitive inhibitors, such as WYE354, pp242, NVP-BEZ235, and Torin1 (Fig. S4); however, this concentration is ∼1,000× higher than that used here for PEL. With PEL being among the most mTOR-addicted lymphomas, the low IC_50_ for MLN0128 is consistent with primary inhibition of only mTOR kinase. These results establish that MLN0128 inhibits mTORC1 and mTORC2 activity without feedback activation of AKT.

10.1128/mBio.02871-18.4 FIG S4 Kinome tree depiction of (A) MLN0128 and (B) other ATP-competitive inhibitor targets in protein kinases, generated using DiscovRx TREEspot version 4. The screen for (A) MLN0128 was performed at 25, 250, 1,000, and 10,000 nM, while the screen for (B) WYE354, pp242, BEZ235, and Torin 1 was performed at 10,000 nM. Data for WYE354, pp242, and Torin 1, were obtained from reference 37. Only kinases with an *S* score of <5% relative to the DMSO control are shown. The *S* score indicated the relative selectivity properties of the drugs, with smaller *S* values signifying a more selective compound. The sizes of the red circles are proportional to the strength of the binding; larger circles imply higher affinity. The full data set is available in Table S1. Download FIG S4, DOCX file, 1.2 MB. Copyright © 2019 Caro-Vegas et al. 2019 Caro-Vegas et al. This content is distributed under the terms of the Creative Commons Attribution 4.0 International license.

10.1128/mBio.02871-18.8 TABLE S1 Complete data set of the kinome study. Download Table S1, XLSX file, 0.06 MB. Copyright © 2019 Caro-Vegas et al. 2019 Caro-Vegas et al. This content is distributed under the terms of the Creative Commons Attribution 4.0 International license.

### MLN0128 inhibits PEL growth *in vivo*.

**FIG 4:**
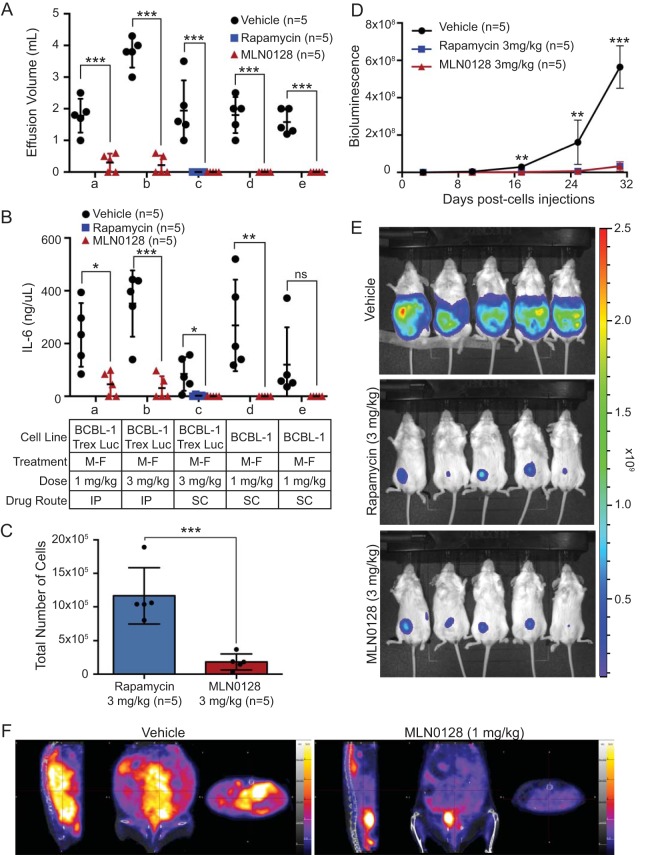
MLN0128 inhibits PEL growth in xenograft models. PEL xenograft models were treated with vehicle (*n* = 25 total), rapamycin (*n* = 5 total), or MLN0128 (*n* = 25 total) at the indicated concentration by i.p. or s.c. injections following a Monday to Friday dosing schedule for 4 weeks for BCBL-1TrexRTA-Luc or 8 weeks for BCBL-1 studies. In each of the experiments a to e, *n* = 5 for each treatment group. MLN0128-treated mice remained active and displayed no toxicity signs such as weight loss. (A) Effusions were harvested and quantified at the end of the study, and (B) IL-6 levels were measured by ELISA (unpaired 2-tailed *t* test; ns, not significant, *, *P* < 0.05, **, *P* < 0.01, and ***, *P* < 0.001, vehicle [*n* = 5] versus MLN0128 [*n* = 5] group or vehicle [*n* = 5] versus rapamycin [*n* = 5] per experiments a to e). (C) PEL cell quantification of PBS cavity wash from mice with no effusion from experiment c (unpaired 2-tailed *t* test; *, *P* < 0.05, **, *P* < 0.01, and ***, *P* < 0.001, MLN0128 [*n* = 5] versus rapamycin [*n* = 5] group). (D) Representative bioluminescent quantification of BCBL-1TrexRTA-Luc studies. (E) Representative image of bioluminescence imaging of BCBL-1TrexRTA-Luc studies and (F) PET-CT of BCBL-1 studies.

To determine *in vivo* efficacy, we used an established xenograft model for PEL (5) augmented by live imaging. Unlike most other lymphomas, PEL grow as effusions in patients. Hence, an intraperitoneal (i.p.) model mimics the clinical presentation. MLN0128 reduced PEL progression of BCBL-1 and BCBL-1TrexRTA-Luc cells, as measured by effusion volume (Fig. 4A) and IL-6 levels (Fig. 4B). There was no significant difference between MLN0128 and rapamycin treatment (Fig. 4A). To study this further, in mice that no longer had overt effusions at the end of the observation period, the cavity was washed with phosphate-buffered saline (PBS) to collect any cells that may still be present on the cavity. Live cells were quantified and showed a significant difference between mice treated with rapamycin and those treated with MLN0128 (Fig. 4C). Weekly bioluminescence measurements showed a significant decrease in region of interest (ROI) luminescence with treatment (Fig. 4D and E; see Fig. S5A and S5B in the supplemental material). To confirm these results, positron emission tomography-computed tomography (PET-CT) was performed. This showed a significant decrease in tumor metabolism between the treated and vehicle groups normalized to pretreatment conditions (Fig. 4F; see Fig. S5C to S5E and Movies S1 and S2 in the supplemental material). To verify that the effusion contained PEL cells rather than mouse cells, we performed Giemsa staining, which showed cells within the effusion had PEL-like features, such as a prominent pleomorphic nucleoli and increased size compared to mouse cells (see Fig. S5F to S5H in the supplemental material). MLN0128 did not cause mortality or evident toxicity, demonstrated by constant body weight (see Fig. S6 in the supplemental material); the treated mice remained active throughout the experiment. This demonstrates that MLN0128 inhibits PEL *in vivo*.

10.1128/mBio.02871-18.5 FIG S5 (A and B) *In vivo* bioluminescent imaging of mice injected with BCBL-1TrexRTA-Luc PEL cells expressing luciferase. Mice were anesthetized, and luminescence was imaged after i.p. injection with d-luciferin. Mice were imaged once a week, starting 3 days after the injection of PEL cells. (C to E) Quantitative PET-CT measurements of FDG uptake by PEL tumors. BCBL-1 cells were intraperitoneally injected, and mice were treated from Monday through Friday with 1 mg/kg MLN0128 (*n* = 5) or vehicle (*n* = 5). Shown is a histogram of the standard uptake value (SUV) within the intraperitoneal region of interest (ROI) (60 bins, from 0 to 3 SUV) for PET-CT imaging (A) 1 week before cell injection to obtain background measurement (*n* = 10) and (B) 6 weeks after treatment with MLN0128 (*n* = 5) or vehicle (*n* = 5). (C) Statistical analysis of vehicle (*n* = 5) and MLN0128 (*n* = 5) groups based on gut ROI SUV per muscle ratio normalized to the control. Data represent the mean ± SD from *n* = 5 animals per experimental group (Student’s *t* test; *, *P* < 0.05, **, *P* < 0.01, and ***, *P* < 0.001, MLN0128 versus vehicle group). (F to H) Representative Giemsa stain of peritoneal effusions from untreated mice. Effusions were spun into cytospin slides and stained with Giemsa solution. Download FIG S5, DOCX file, 1.0 MB. Copyright © 2019 Caro-Vegas et al. 2019 Caro-Vegas et al. This content is distributed under the terms of the Creative Commons Attribution 4.0 International license.

10.1128/mBio.02871-18.6 FIG S6 Effect of MLN0128 treatment on mice body weight. Shown are the body weights of mice injected intraperitoneally with (A to C) BCBL-1TrexRTA-Luc and (D and E) BCBL-1 cells treated with MLN0128 (1 to 3 mg/kg), rapamycin (3 mg/kg), or vehicle (20% DMSO). Changes in body weight were monitored through the course of each study. Data represent the mean ± SD from *n* = 5 animals per experimental group. Download FIG S6, DOCX file, 0.2 MB. Copyright © 2019 Caro-Vegas et al. 2019 Caro-Vegas et al. This content is distributed under the terms of the Creative Commons Attribution 4.0 International license.

10.1128/mBio.02871-18.9 MOVIE S1 PET-CT imaging of a representative mouse in the vehicle-treated group. Download Movie S1, MOV file, 0.8 MB. Copyright © 2019 Caro-Vegas et al. 2019 Caro-Vegas et al. This content is distributed under the terms of the Creative Commons Attribution 4.0 International license.

10.1128/mBio.02871-18.10 MOVIE S2 PET-CT imaging of a representative mouse in the MLN-treated group. Download Movie S2, MOV file, 0.8 MB. Copyright © 2019 Caro-Vegas et al. 2019 Caro-Vegas et al. This content is distributed under the terms of the Creative Commons Attribution 4.0 International license.

### MLN0128 induces apoptosis in rapamycin-resistant PEL.

**FIG 5:**
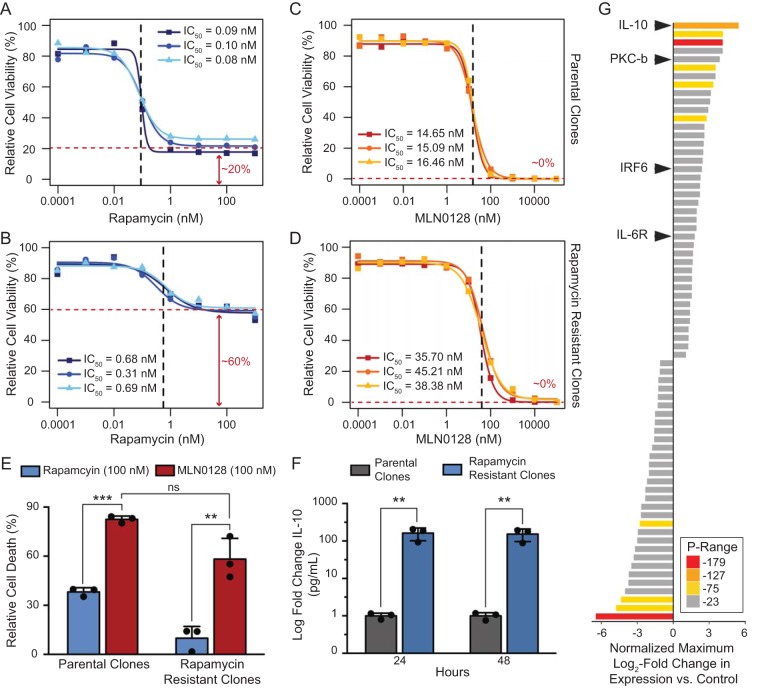
MLN0128 induces apoptosis of rapamycin-resistant clones. (A and C) Parental and (B and D) rapamycin-resistant clones were treated with increasing concentrations of (A and B) rapamycin or (C and D) MLN0128 for 48 h, and cell viability was measured by CellTiter-Glo luminescent cell viability assay. Dose-response curves were generated as a percentage of the vehicle (100%) and the no-cell control (0%) in R. The IC_50_s (95% confidence interval) are shown by panel as follows: (A and B) rapamycin treatment of (A) the parental clones, 1, 0.09 ± 0.04, 2, 0.10 ± 0.03, and 3, 0.08 ± 0.02 nM; (B) rapamycin-resistant clones, 1, 0.68 ± 0.75, 2, 0.31 ± 0.24, and 3, 0.69 ± 0.88 nM; (C and D) MLN0128 treatment of (C) the parental clones, 1, 14.65 ± 3.10, 2, 15.09 ± 2.80, and 3, 16.46 ± 5.25 nM; and (D) rapamycin-resistant clones, 1, 35.70 ± 5.50, 2, 45.21 ± 8.90, and 3, 38.39 ± 7.82 nM. Data represent the mean from *n* = 4 independent wells. (E) Parental and rapamycin-resistant clones were treated with 100 nM rapamycin or MLN0128 for 48 h. Cell apoptosis was measured by annexin V FACS; the percentages indicate annexin V-positive cells (apoptotic) normalized to the control. (F) Levels of IL-10 were measured by ELISA in parental and RR clones after 24 or 48 h of seeding time. Data represent the mean ± SD from *n* = 3 independent clones (unpaired 2-tailed *t* test; ns, not significant, **, *P* < 0.01, and ***, *P* < 0.001, rapamycin versus MLN0128 group). (G) The RNA profile of parental and rapamycin-resistant clones was obtained by RNA-seq analysis.

To test the hypothesis that MLN0128 was active against rapamycin-resistant (RR) PEL, we generated RR PEL, by culturing BCBL-1TrexRTA-Luc in increasing concentrations of rapamycin for 3 months, followed by colony formation assay and single-colony isolation. MLN0128 did not yield any colonies under the same schedule. Each of three RR clones exhibited a 6-fold increase in rapamycin IC_50_ compared to parental cells (Fig. 5A and B). The surviving fraction increased ∼20% to ∼60% in the RR compared to the parental clone. Even though the MLN0128 IC_50_ had a 2-fold increase between the parental and RR clones, MLN0128 was still effective at decreasing PEL viability to 0% in all RR clones (Fig. 5C and D). Additionally, MLN0128 induced apoptosis in both RR and parental clones to a similar degree (Fig. 5E). Exome sequencing of both parental and RR clones did not uncover mutations in the genes coding for mTOR or FKBP1 or other known genes in the mTOR pathway. To test whether transcriptional changes correlated with rapamycin resistance, we performed transcriptome sequencing (RNA-seq). Principal-component analysis uncovered significant and consistent differences between three parental and three RR clones (see Fig. S7A in the supplemental material). The most consistent change was the upregulation of IL-10 in the RR clones (Fig. 5G; see Fig. S7B in the supplemental material). This was also reflected at the protein level since the RR clones exhibited an ∼100-fold increase of secreted IL-10 by comparison to the parental clones (Fig. 5F). In sum, MLN0128 is efficacious even against RR PEL.

10.1128/mBio.02871-18.7 FIG S7 (A) Principal-component analysis (PCA) of rapamycin-resistant clones (*n* = 3) displayed as triangles and parental clones (=3) displayed as circles. Shown is total 2D PCA mapping represent 87% of variance (PC1 = 67% and PC2 = 20%). (B) Volcano plot displaying genes differentially expressed between rapamycin-resistant and parental clones. The *y* axis corresponds to the mean expression value of log_10_ (*P* value), and the *x* axis displays the log_2_ fold change value. The red dots represent the most significant (*P* < 1 × 10^−6^) transcripts with differential expression between rapamycin-resistant and parental clones. The gray dots represent the nonsignificant transcripts (*P* > 1 × 10^−6^) between rapamycin-resistant and parental clones. Download FIG S7, DOCX file, 0.3 MB. Copyright © 2019 Caro-Vegas et al. 2019 Caro-Vegas et al. This content is distributed under the terms of the Creative Commons Attribution 4.0 International license.

## DISCUSSION

PEL is a particularly aggressive type of post-germinal center B cell lymphoma, which is caused by KSHV and which manifests as liquid effusions in body cavities. Prognosis is poor, and resistance to conventional chemotherapy develops quickly. A defining feature of PEL is their dependence on PI3K/Akt/mTOR signaling, which seems to be driven by viral proteins rather than activating mutations in this pathway. Consequently, mTOR inhibitors such as rapamycin (5) and PI3K/mTOR inhibitors such as NVP-BEZ235 (6) show preclinical efficacy. Unfortunately, NVP-BEZ235 was too toxic clinically (39, 40), and rapamycin was cytostatic rather than cytotoxic for PEL. While this suffices for rapamycin to control KS (24, 26) and to yield a clinical benefit in mantle cell lymphoma and renal cancer, it limits its use against a highly aggressive lymphoma such as PEL. Of note, the orally bioavailable rapamycin derivative everolimus was clinically efficacious against advanced renal cell carcinoma patients, who progressed on pan-receptor tyrosine kinase inhibitors, such as sunitinib or sorafenib (41). This is consistent with mTOR being downstream of vascular endothelial growth factor (VEGF) receptors, which are targeted by broad-spectrum receptor tyrosine kinase inhibitors, and which are known oncogenic drivers in PEL and KS.

MLN0128 is an orally bioavailable, ATP-competitive inhibitor that targets mTORC1 and mTORC2. It showed promise in preclinical studies and has passed phase 1 trials in multiple myeloma, non-Hodgkin’s lymphoma, and Waldenström’s macroglobulinemia (32). This study established preclinical efficacy and mechanism of action for MLN0128 in PEL. MLN0128 had an IC_50_ of 10 to 50 nM across multiple PEL cell lines (Table 1). This effective concentration is in range of currently approved cytotoxic agents for PEL (e.g., doxorubicin), as well as rapamycin/everolimus. While there are many targeted agents under consideration against KSHV-associated cancers, the only ones that demonstrated some clinical efficacy in KS had similar low nanomolar concentration IC_50_s in the PEL culture system. In the absence of a KS tumor model, inhibition of PEL growth in culture and in xenografted mice represents the most stringent preclinical model for testing novel agents.

Importantly, the action of MLN0128 was effective against a doxorubicin-resistant PEL cell line, BCP-1: i.e., MLN0128 was independent of p53 mutation status in PEL (42). MLN0128 induced apoptosis, whereas rapamycin and its analogs only induced cell cycle arrest (Fig. 2). MLN0128 effectively inhibited mTORC1 and mTORC2 activity, while rapamycin caused a transient increase in Akt S473 phosphorylation, due to a feedback loop, indicating the presence of active mTORC2 (31) (Fig. 3). There are various models that may explain the greater efficacy of MLN0128. Most likely, the dual inactivation of both mTORC1 and mTORC2 by MLN0128 might explain its greater effect on PEL compared to rapamycin. This would also disrupt the compensatory upregulation of mTORC2 in response to mTORC1 inhibition as observed in solid tumors. Another explanation could be that the allosteric inhibitor rapamycin only inhibited some of the downstream pathways of mTORC1, whereas the ATP-competitive mTORC inhibitors tend to have a far broader effect on mTORC1 substrates (30). A third possibility could involve differential intracellular accumulation and pharmacology between rapamycin derivatives and MLN0128. Lastly, rapamycin has been shown to inhibit KSHV reactivation, specifically replication and transcription activator (RTA) function (43). We have not tested it, but presumably MLN0128 would do so as well and more completely. This in turn may downregulate RTA-dependent oncoproteins of KSHV such as vGPCR (12, 13) or the viral protein kinase orf36, which is a functional homolog of p70S6K (44). As PEL (and KS) are extraordinarily sensitive to mTORC inhibitors, the detailed study of these compounds in PEL may yield further mechanistic insights into their mechanism of action.

MLN0128 was active *in vivo* at 1 mg/kg of body weight/day (Fig. 4) and showed no toxicity. As previously noted, PEL develops resistance to rapamycin overtime. In culture, we were able to shift the IC_50_ for rapamycin by 100-fold within 3 months, while under the same condition, resistance to MLN0128 only changed 2-fold. The differential effect in PEL may be explained by the cytostatic activity of rapamycin, which gives PEL the chance to develop resistance. On the other hand, MLN0128 induces rapid apoptosis and therefore makes the development of resistance more difficult. Of note, we did not recover any previously described mutations in the RR clones, such as mutations in FKBP. Our screen was limited as we only sequenced a few resistant isolates. At the same time, all the RR clones tested had an upregulation of IL-10 at the transcriptional and protein levels. This was consistent with the rapid adaptation in culture, rather than selection for resistance mutants. It may elucidate a mechanism of rapamycin resistance analogous to the role IL-7 plays in mediating resistance to rapamycin in preclinical models of B cell leukemia (45). Importantly, MLN0128 was active against RR PEL (Fig. 5). In toto, these studies establish the mechanism of action for MLN0128 in this lymphoma and suggest that clinical studies of MLN0128 in PEL and KS are warranted.

## MATERIALS and METHODS

### Compounds and cell culture.

MLN0128 and rapamycin were purchased from Selleckchem. Compounds were dissolved in DMSO (Sigma-Aldrich), aliquoted, and stored at −80°C. Cell lines were cultured in RPMI 1640 supplemented with 100 U/ml penicillin-streptomycin (Thermo Fisher), 2 mM l-glutamine (Thermo Fisher), and 10% fetal bovine serum (FBS) (Sigma-Aldrich), maintained at 37°C in 5% CO_2_, and passaged for no more than 3 months. BC-1, BCBL-1, BC-3, and BCP-1 were obtained from ATCC. BCBL-1TrexRTA cells were a gift from J. Jung (46). RedFect (Perkin Elmer) was used to confer continuous red-luciferase expression (BCBL-1TrexRTA-luc), and cells were maintained in hygromycin B (20 µg/ml) and puromycin (1.25 µg/ml). Cell lines were authenticated by NextGen-based HLA and short tandem repeat (STR) typing (https://www.med.unc.edu/vironomics) and found free of mycoplasma by the Mycoalert mycoplasma test kit (Lonza).

### Cell proliferation and cell viability.

Cells were cultured at indicated concentration of inhibitor and counted in octuplicates using trypan blue (Sigma). Cell viability was determined at 48 h by CellTiter-Glo Assay (Promega) as per the manufacturer’s instructions. Luminescence was measured at 560 nm using FLUOstar Optima (BMG Lab Tech). IC_50_s were calculated using R.

### Colony formation.

Cells were plated in triplicates in 10% FBS complete RPMI medium with 1% methylcellulose containing the indicated concentration of inhibitor. Colonies were counted after 2 weeks using a Leica MZ 6 microscope.

### Apoptosis and cell cycle.

Cell were treated the indicated concentration of inhibitor and incubated for 24, 48, 72, and 96 h. For the apoptosis assay, cells were washed in ice-cold PBS and resuspended in binding buffer containing annexin V and propidium iodide (Life Technologies). For the cell cycle assay, cells were washed with cold PBS, fixed with cold 100% ethanol, treated with RNase A, and stained with 10 µg/ml propidium iodide in PBS. A total of 100,000 events were collected on MacsQuant VYB (Miltenyi Biotec) and analyzed using FlowJo v10.1 (Tree Star).

### Immunoblotting.

Cells were treated with indicated concentration of inhibitors and lysed in radioimmunoprecipitation assay (RIPA) buffer supplemented with protease inhibitor cocktail (Roche), 30 mM β-glycerol phosphate, 50 mM sodium fluoride, and 1 mM sodium orthovanadate, incubated for 1 h on ice, and centrifuged at 14,000 × *g* for 5 min at 4°C. The protein concentration was determined by bicinchoninic acid (BCA) assay, and equal amounts of protein were loaded onto 10% SDS-polyacrylamide gel, separated by electrophoresis, and transferred to a polyvinylidene difluoride (PVDF) membrane (Sigma-Aldrich). The following primary antibodies were used at a 1:1,000 dilution: phospho-S6 ribosomal protein Ser240–244 (no. 2215), S6 ribosomal protein 5G10 (no. 2217), phospho-4E-BP1 Ser65 (no. 9451), 4E-BP1 (no. 9452), phospho-AKT Ser473 D93 XP (no. 4060), AKT pan-11E7 (no. 4685), phospho-NDGR1 Thr346 (no. 3217), NDRG1 D6C2 (no. 9408), cleaved caspase-3 Asp175 (no. 9664), and cleaved PARP Asp214 D64E10 XP (no. 5625) from Cell Signaling and β-actin (A5441) from Sigma. Signal was detected with horseradish peroxidase (HRP)-conjugated secondary antibodies (Vector Labs) and developed using ECL enhanced chemiluminescence substrate (Pierce). Phosphorylated proteins were detected first, and then membranes were stripped with One Minute Advance Western blot stripping buffer (GM Biosciences) and probed for total protein.

### ELISA.

For enzyme-linked immunosorbent assay (ELISA), IL-6 and IL-10 were quantified using the Ready-Set-Go kit (eBioscience) per the manufacturer’s instructions. Plates were washed with plate washer ELx405 (Biotek). Absorbance was measured at 450 nm using spectrophotometer Infinite M200 PRO (Tecan).

### Kinome scan.

DiscoveRx 442 kinome-wide selectivity profiling was conducted by DiscoveRx Bioscience with KinomeScan technology.

### Xenograft studies.

Six-week-old female NSG mice (Jackson Laboratory) were injected intraperitoneally (i.p.) with 1 × 10^5^ cells. After 3 days, mice were randomized to a vehicle or treatment group, based on initial luminescent results before drug injections. MLN0128 and rapamycin were administered by i.p. or subcutaneous (s.c.) injections as indicated. The entire study group was euthanized when the vehicle group body score dropped below 2 or >20% loss of body weight. Effusion volumes were collected from the peritoneal cavity and measured. In mice that did not have any effusion to be collected, the cavity was washed with PBS to obtained PEL cells still present on the cavity. Effusions were diluted 1:3 with cold PBS with 2% FBS and centrifuged at 300 × *g* for 5 min, and supernatant and cell pellets were flash frozen and stored at −80. For every experiment, *n* = 5 for each treatment group, based on power calculations to record statistically significant (*P* ≤ 0.05) alterations for individual measures. All mouse studies were performed by the UNC Lineberger Animal Studies Core Facility; the investigator was not present during group randomization or outcome assessment of bioluminescent measurements and fluid collection.

### Bioluminescent imaging.

Bioluminescent imaging was performed using the Xenogen IVIS-Lumina system (Caliper Life Sciences). Mice were anesthetized using 2% isoflurane and 100% oxygen at a flow rate of 2.5 liters/min. Then 10 μl/g of a 15-mg/ml sterile d-luciferin firefly substrate (Biosynth International, Inc.) dissolved in PBS was administered by i.p. injection, and 15 min after substrate injection, the mice were imaged for up to 2 min. Each image was saved for subsequent analysis. The images were analyzed with Living Image 4.2. The scales to the right of the images in Fig. 4D represent the photon emission from the tissue surfaces and are expressed as photons per second per centimeter squared per steradian (p/s/cm^2^/sr).

### PET-CT imaging.

MicroPET and micro-CT images were acquired with an eXplore Vista small animal PET-CT scanner (GE Healthcare) with a center resolution of 1.2 mm and a 46-mm axial field of view. Food was removed from mouse cages at least 4 h before radiotracer injection. Mice were anesthetized in an induction chamber with a 3% isoflurane–oxygen mixture (vol/vol) and then injected intravenously via tail vein catheter with approximately 300 µCi of ^18^F-labeled fluorodeoxyglucose ([^18^F]FDG). The syringe and catheter were then removed, and residue activity was measured and subtracted from the total injected activity. After the injection, mice were allowed to recover from anesthesia and placed back in the cage to resume activity. Approximately 45 min after injection of the radiotracer, mice were placed back in the induction chamber with 3% isoflurane, which was reduced to 1.5% for maintenance of anesthesia before the animal was placed prone on the PET cradle, with legs secured to the side. A respiratory monitor (SA Instruments, Inc., Stony Brook, NY) was placed above the animal. Temperature was monitored with an infrared thermometer and maintained with a heat lamp. A CT scan of the animal’s abdominal region was then taken for attenuation correction and anatomical reference, with a tube current of 140 µA and voltage of 40 kVp. Sixty minutes after the injection of the radiotracer, a 20-min static PET scan of the abdominal region was acquired. PET emission data were corrected for decay/dead time and reconstructed with the Vista software, using a 2D ordered subset expectation maximization (2D-OSEM) algorithm that corrects for random coincidences, scatter, and attenuation and calibrates the image to standardized uptake value (SUV).

### Drug resistance.

Cell lines resistant to rapamycin (RR1, RR2, and RR3) were generated by exposing the parental PEL cell line, BCBL-1TrexRTA-Luc, to an increasing dose of rapamycin (up to 1,000 nM/100× IC_50_) for 3 months. Clones were obtained by extracting and expanding a single colony from a colony formation assay. Clones were maintained at 100 nM rapamycin.

### Exome sequencing and mutation calling analysis.

Total nucleic acid was extracted from 1 × 10^6^ cells using a MagNA Pure compact nucleic acid isolation kit I large-volume kit (Roche) and quantitated by Qubit 3.0 double-stranded DNA (dsDNA) high-sensitivity (HS) assay (Life Technologies). Barcoded exome sequencing libraries were prepared from 100 ng DNA with an Ion AmpliSeq Exome RDY library preparation kit (Life Technologies) using protocol MAN00009808 Rev: A.0. Libraries were quantitated by Qubit dsDNA HS assay, sized with an Agilent Bioanalyzer 2100 high-sensitivity DNA assay (Agilent Technologies), and pooled to 80 pM final concentration. Templating and loading onto the Ion 540 Chip (Life Technologies) were automated on the Ion Chef (Life Technologies). Samples were sequenced on the Ion S5 system (Life Technologies). Base calling, quality filtering, and demultiplexing were performed on the Ion S5 (Life Technologies) with default parameters. Raw reads were trimmed to >50 bp and mapped to the human genome (NCBI build hg38_2016) using CLC Genomics Workbench 9 (CLC bio). The CLC basic variant detection tool was used with the parameters minimum average quality score = 19, minimum frequency = 90%, and minimum coverage = 40. Only single-nucleotide polymorphism (SNP) calls with forward and reverse balance of 0.25 < x < 0.75 were included in the analysis.

### RNA-seq and analysis.

Total RNA was purified and poly(A) enriched using Oligotex mRNA minikits (Qiagen). Library preparation was performed according to the Ion total RNA-Seq kit v2 whole-transcriptome RNA protocol (ThermoFisher Scientific, publication no. MAN0010654, revision B.0) with the following modifications: purification of fragmented RNA and cDNA was with 1.8× Agencourt RNAClean XP beads (Beckman Coulter, Inc.) (*Ovation Universal RNA-Seq System User Guide*; NuGEN), and an additional Agencourt AMPure XP 1.0× bead (Beckman Coulter, Inc.) cleanup of the finished library was performed. Libraries were quality controlled and quantitated using the 2100 high-sensitivity DNA assay (Agilent Technologies) and Qubit dsDNA HS assay (ThermoFisher Scientific). A 50 pM concentration of library was templated on the Ion Torrent Chef for 200 bp and sequenced on the Ion Torrent S5 using a 540 chip. Subsequent steps included quality control using bbduk version 37.25 (k = 23 mink = 11 ktrim=r hdist = 1 minlength = 100 qtrim=rl trimq = 20 ftl = 10 ftr = 600 maq = 20 tpe tbo), mapping to reference genome (GRCh38, STAR aligner v2.5.3a, gencode v22 annotations), with read counting on genes (summarizeOverlaps, mode: intersection-strict, singleEnd). Differential gene expression was calculated using DESeq2 working with a simple interaction term for the model: design = ∼ Drug + Clone.Number. PCA, distance heat maps, and other figures were generated in R using DESeq2 and ggplot2. Genes were considered differentially expressed by the Wald test, with an adjusted (Benjamini-Hochberg) *P* value of <0.05.

### Statistics.

Results are reported as mean ± standard deviation (SD). All cellular experiments were repeated in at least three complete biological replicates. The unpaired 2-tailed *t* test with Welch correction (do not assume equal SD) was used to statistically compare groups.

### Code availability.

For drug-response curves, the public DRC package version 3.0 from R was used. The package is available at https://cran.r-project.org/web/packages/drc/index.html.

### Study approval.

Studies were approved by the IACUC of the University of North Carolina, Chapel Hill, NC.

### Data availability.

Raw sequences have been deposited under NCBI BioProject accession no. PRJNA414221. The complete code for analysis and gene list have been deposited in bitbucket (https://ddittmer@bitbucket.org/dittmerlab/rapamycin_mln0128_resistance_rnaseq.git).
