# Supplementary material for: Targeting mTOR with MLN0128 Overcomes Rapamycin and Chemoresistant Primary Effusion Lymphoma
Source: mBio. 2019 Feb 19;10(1):e02871-18. doi: 10.1128/mBio.02871-18 (PMC6381283; doi:10.1128/mBio.02871-18)
Supplement: FIG S1 [file mBio.02871-18-sf001.docx]

**Supplemental Figures**


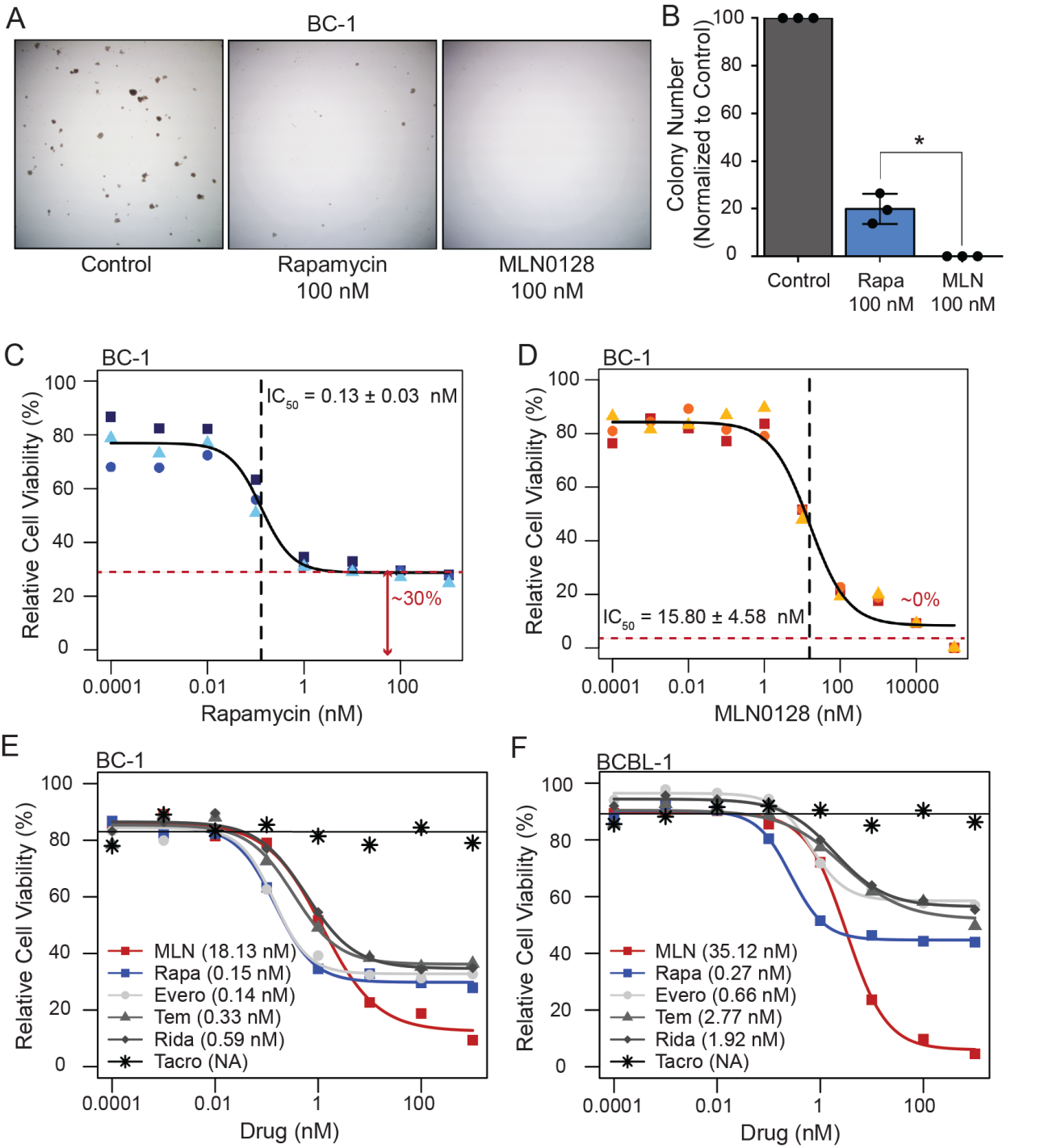


**Supplemental Figure 1:** (A, B) BC-1 cells were incubated with 100 nM of rapamycin or MLN0128, inhibition of cell proliferation was assessed by colony formation assay after 2 weeks. Data represents the mean ± SD of n=3 independent experiments (Student t-test, *p < 0.05, **p < 0.01 rapamycin vs MLN0128 group). BC-1 cells were treated with increasing concentrations of (C) rapamycin or (D) MLN0128 for 48 hrs, cell viability was measured by CellTiter-Glo® Luminescent Cell Viability Assay. (E) BC-1 and (F) BCBL-1 cells were treated with increasing concentrations of MLN0128 (MLN), rapamycin (rapa), everolimus (evero), temsirolimus (tem), ridaforolimus (rida) and tacrolimus (tacro) for 48 hrs, cell viability was measured by CellTiter-Glo® Luminescent Cell Viability Assay. Dose-response curves were generated as a % of the no drug (100%) and no cells control (0%) in R. Data represents the mean of n=4 independent wells.
