# Supplementary material for: Targeting mTOR with MLN0128 Overcomes Rapamycin and Chemoresistant Primary Effusion Lymphoma
Source: mBio. 2019 Feb 19;10(1):e02871-18. doi: 10.1128/mBio.02871-18 (PMC6381283; doi:10.1128/mBio.02871-18)
Supplement: FIG S2 [file mBio.02871-18-sf002.docx]

**
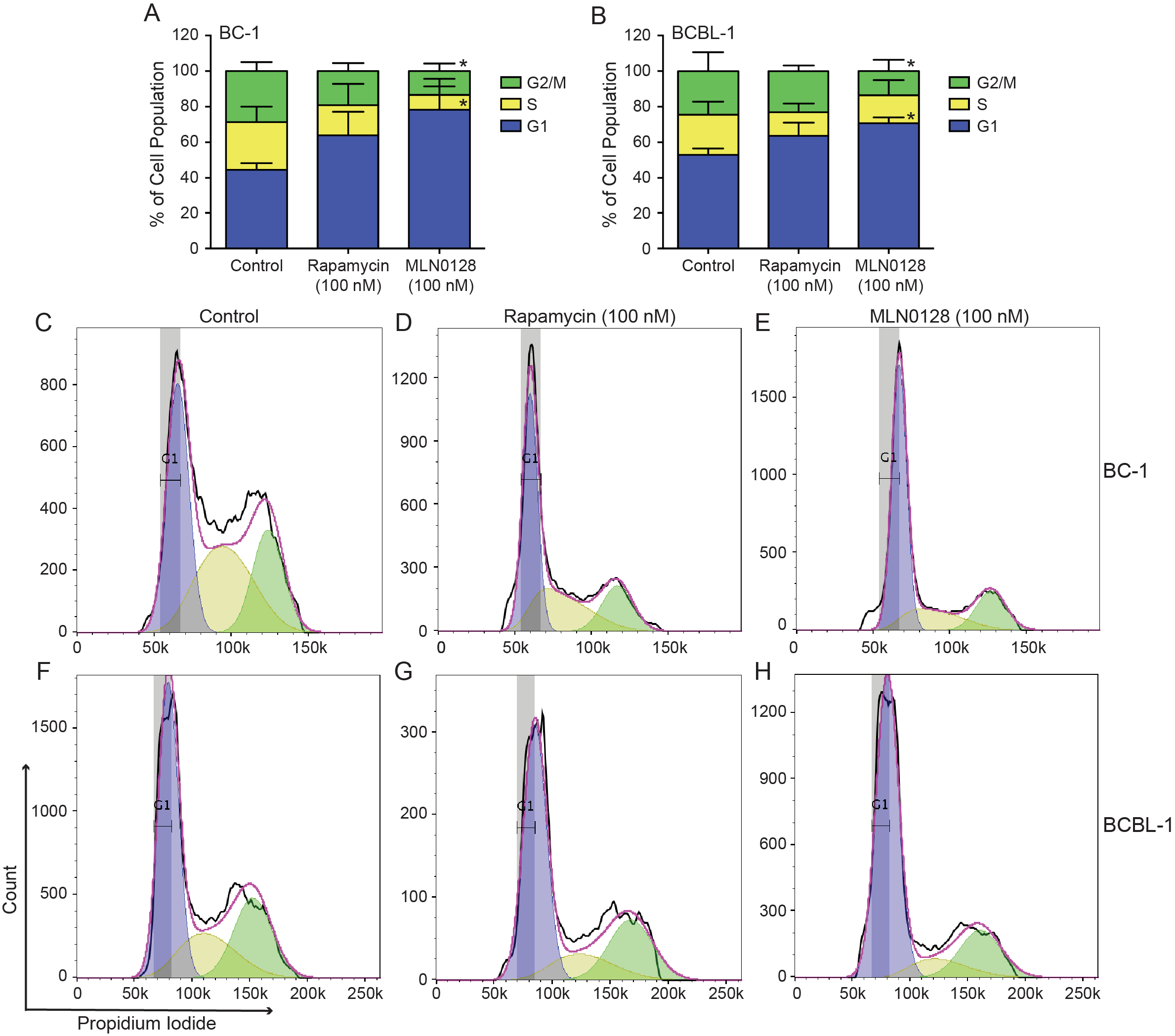
**

**Supplemental Figure 2:** (A) BC-1 and (B) BCBL-1 cells were treated with 100 nM of rapamycin and MLN0128 for 48 hrs. Cells were stained with propidium iodide and analyzed by FACS and FlowJo with the tool of cell cycle distribution. Representative FACS analysis in Flowjo for cell cycle distribution for BC-1 (C-E) and BCBL-1 (F-H), plots are live-gated. Data represents the mean ± SD of n=3 independent experiments (Student t-test, *p < 0.05, **p < 0.01, ***p<0.001 control vs. MLN0128 group).
