# Supplementary material for: Targeting mTOR with MLN0128 Overcomes Rapamycin and Chemoresistant Primary Effusion Lymphoma
Source: mBio. 2019 Feb 19;10(1):e02871-18. doi: 10.1128/mBio.02871-18 (PMC6381283; doi:10.1128/mBio.02871-18)
Supplement: FIG S3 [file mBio.02871-18-sf003.docx]

**
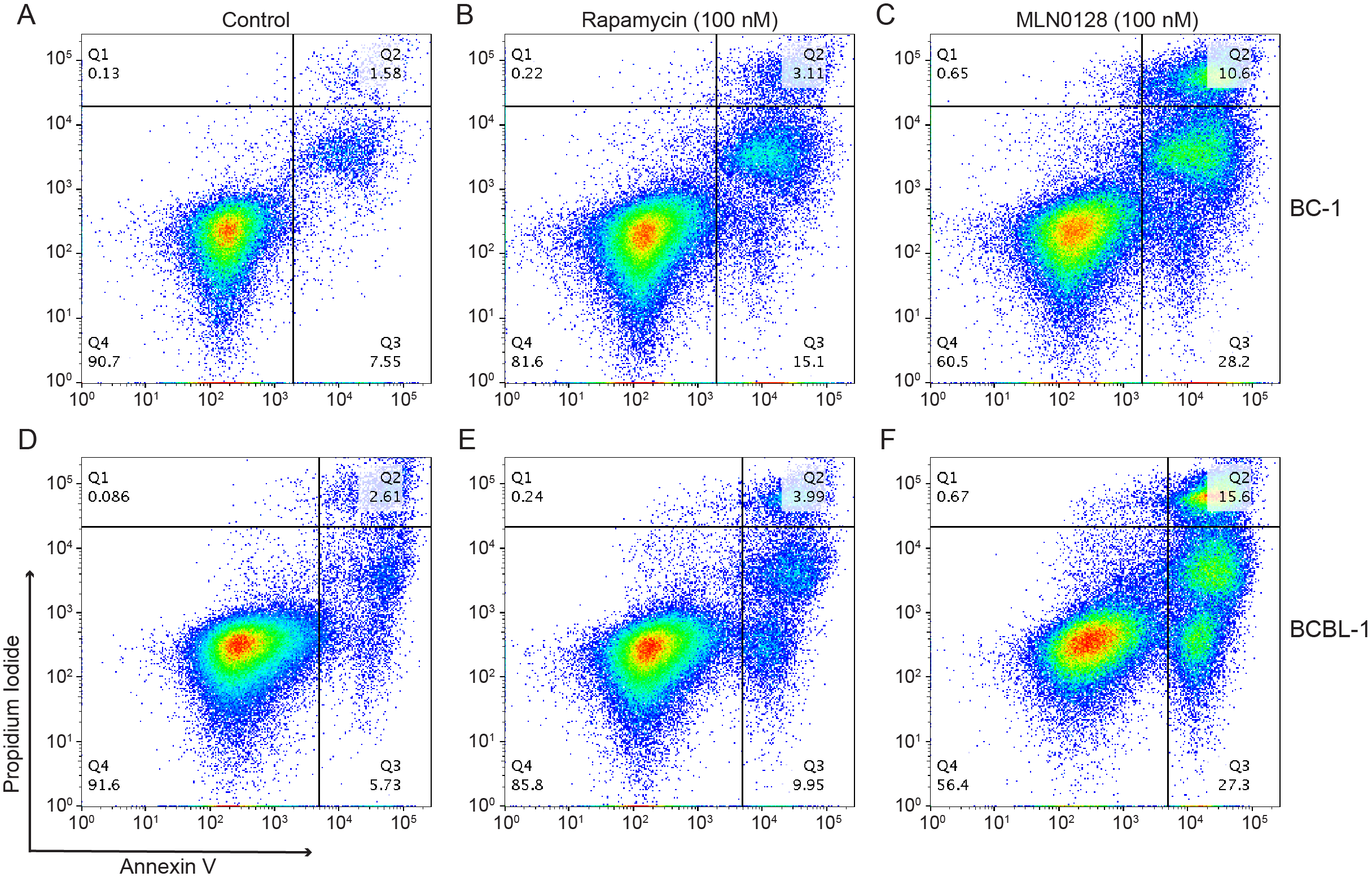
**

**Supplemental Figure 3: Gating strategy for delineating viable, early apoptotic, late apoptotic and necrotic cells.** Representative FACS analysis in FlowJo from annexin V/PI assay. (A-C) BC-1 and (D-F) BCBL-1 cells were treated with 100 nM of rapamycin or MLN0128 as indicated and incubated for 48 hrs. In each panel, the lower left quadrant indicates viable cells (annexin negative, PI negative, Q4), lower right indicates early apoptotic cells (annexin positive, PI negative, Q3), the upper right quadrant indicates late apoptotic cells (annexin positive, PI positive, Q2) and the upper left quadrant indicates necrotic cells (annexin negative, PI positive, Q1).
