# Supplementary material for: Targeting mTOR with MLN0128 Overcomes Rapamycin and Chemoresistant Primary Effusion Lymphoma
Source: mBio. 2019 Feb 19;10(1):e02871-18. doi: 10.1128/mBio.02871-18 (PMC6381283; doi:10.1128/mBio.02871-18)
Supplement: FIG S4 [file mBio.02871-18-sf004.docx]

**
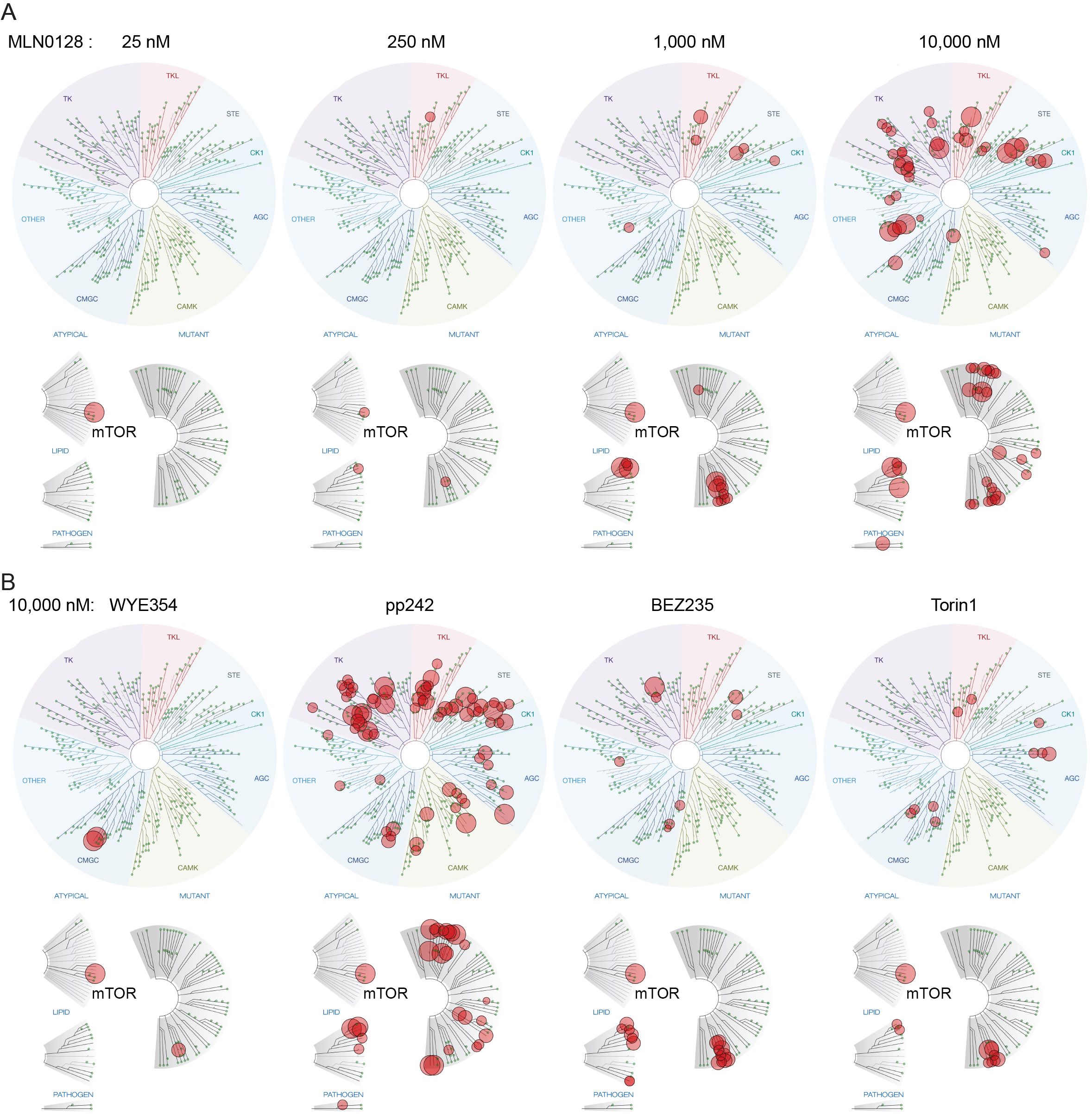
**

**Supplemental Figure 4:** Kinome tree depiction of (A) MLN0128 and (B) other ATP-competitive inhibitors targets in protein kinases, generated using DiscovRx TREEspot Version 4. The screen for (A) MLN0128 was performed at 25, 250, 1,000 and 10,000 nM., while the screen for (B) WYE354, pp242, BEZ235 and Torin 1 was performed at 10,000 nM. Data for WYE354, pp242 and Torin 1, were obtained from (37). Only kinases with a S score of <5% relative to DMSO control are shown. *S* score indicated the relative selectivity properties of the drugs with smaller *S* values signifying a more selective compound. The sizes of the *red circle* are proportional to the strength of the binding; the *larger circles* imply higher affinity. The full dataset is available as supplemental Table 1.
