# Supplementary material for: Targeting mTOR with MLN0128 Overcomes Rapamycin and Chemoresistant Primary Effusion Lymphoma
Source: mBio. 2019 Feb 19;10(1):e02871-18. doi: 10.1128/mBio.02871-18 (PMC6381283; doi:10.1128/mBio.02871-18)
Supplement: FIG S5 [file mBio.02871-18-sf005.docx]

**
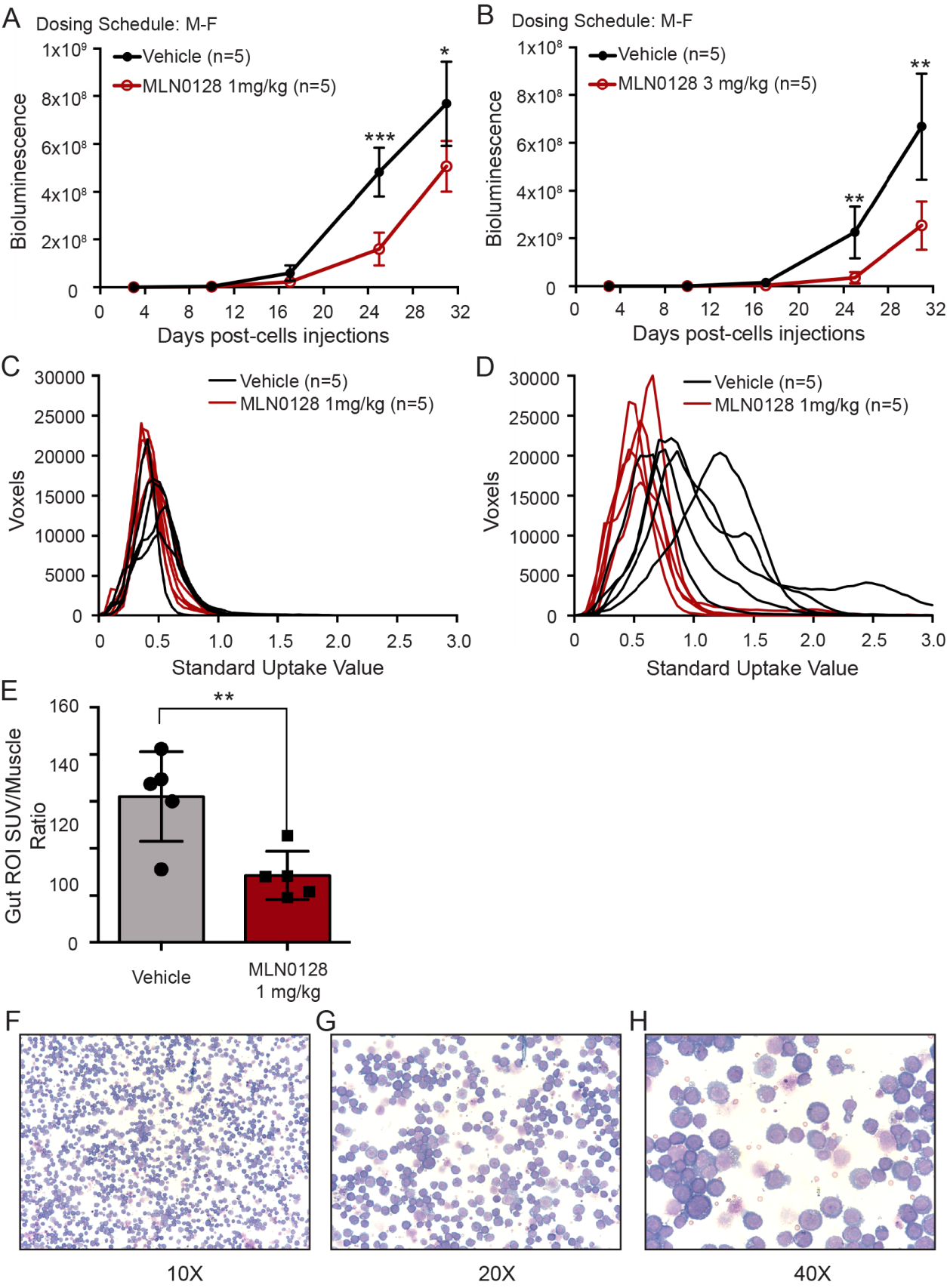
**

**Supplemental Figure 5: (A-B)** In vivo bioluminescent imaging, mice were injected with BCBL-1TrexRTA-Luc, PEL cells expressing luciferase. Mice were anesthetized and luminescence was imaged after IP injection with d-luciferin. Mice were imaged once a week, starting 3 days after the injection of PEL cells. (C-E) Quantitative PET-CT measurements of FDG uptake by PEL tumors. BCBL-1 cells were intraperitoneally injected and mice were treated from M-F with MLN0128 1 mg/kg (n=5) or vehicle (n=5). Histogram of the standard uptake value (SUV) within the intraperitoneal region of interest (ROI) (60 bins, from 0-3 SUV) for PET-CT imaging (A) 1 week before cell injection to obtain background measurement (n=10) and (B) 6 weeks after MLN0128 treatment (n=5) or vehicle (n=5). (C) Statistical analysis of vehicle (n=5) and MLN0128 (n=5) group based on gut ROI SUV per muscle ratio normalized to control. Data represents the mean ± SD of n=5 animals per experimental group (Student t-test, *p < 0.05, **p < 0.01, ***p<0.001 MLN0128 vs vehicle group). (F-H) Representative Giemsa stain of peritoneal effusions from untreated mice. Effusion were spun into cytospin slides and stained with Giemsa solution. Mouse red blood cells are identified by black arrows.
