# Supplementary material for: Targeting mTOR with MLN0128 Overcomes Rapamycin and Chemoresistant Primary Effusion Lymphoma
Source: mBio. 2019 Feb 19;10(1):e02871-18. doi: 10.1128/mBio.02871-18 (PMC6381283; doi:10.1128/mBio.02871-18)
Supplement: FIG S6 [file mBio.02871-18-sf006.docx]

**
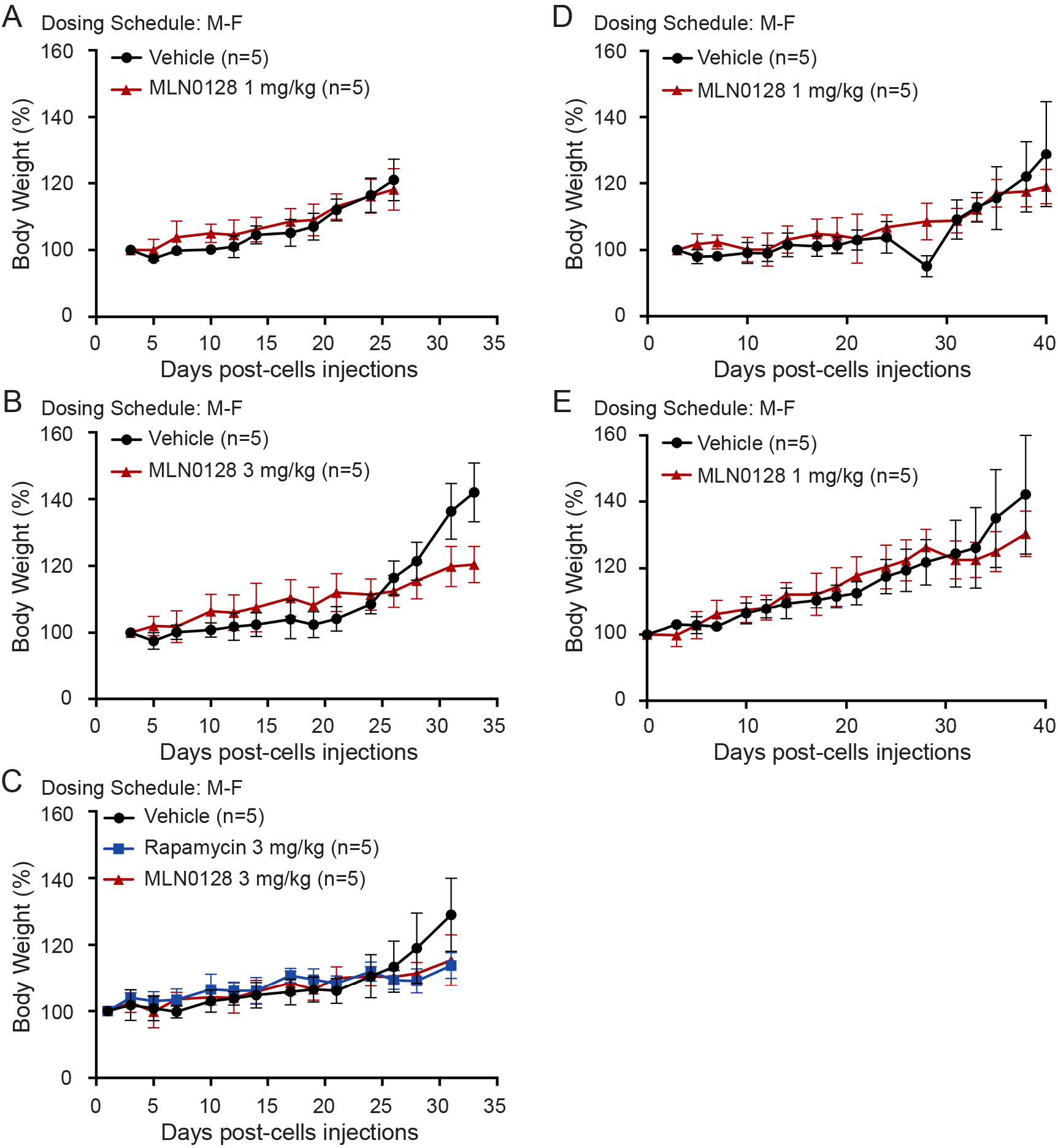
**

**Supplemental Figure 6: Effect of MLN0128 treatment on mice body weight.** Body weight of mice injected intraperitoneally with (A-C) BCBL-TrexRTA-Luc and (D-E) BCBL1 treated with MLN0128 (1-3 mg/kg), rapamycin (3 mg/kg) or vehicle (20% DMSO). Changes in body weight were monitored through the course of each study. Data represents the mean ± SD of n=5 animals per experimental group.
