# Supplementary material for: Targeting mTOR with MLN0128 Overcomes Rapamycin and Chemoresistant Primary Effusion Lymphoma
Source: mBio. 2019 Feb 19;10(1):e02871-18. doi: 10.1128/mBio.02871-18 (PMC6381283; doi:10.1128/mBio.02871-18)
Supplement: FIG S7 [file mBio.02871-18-sf007.docx]

**
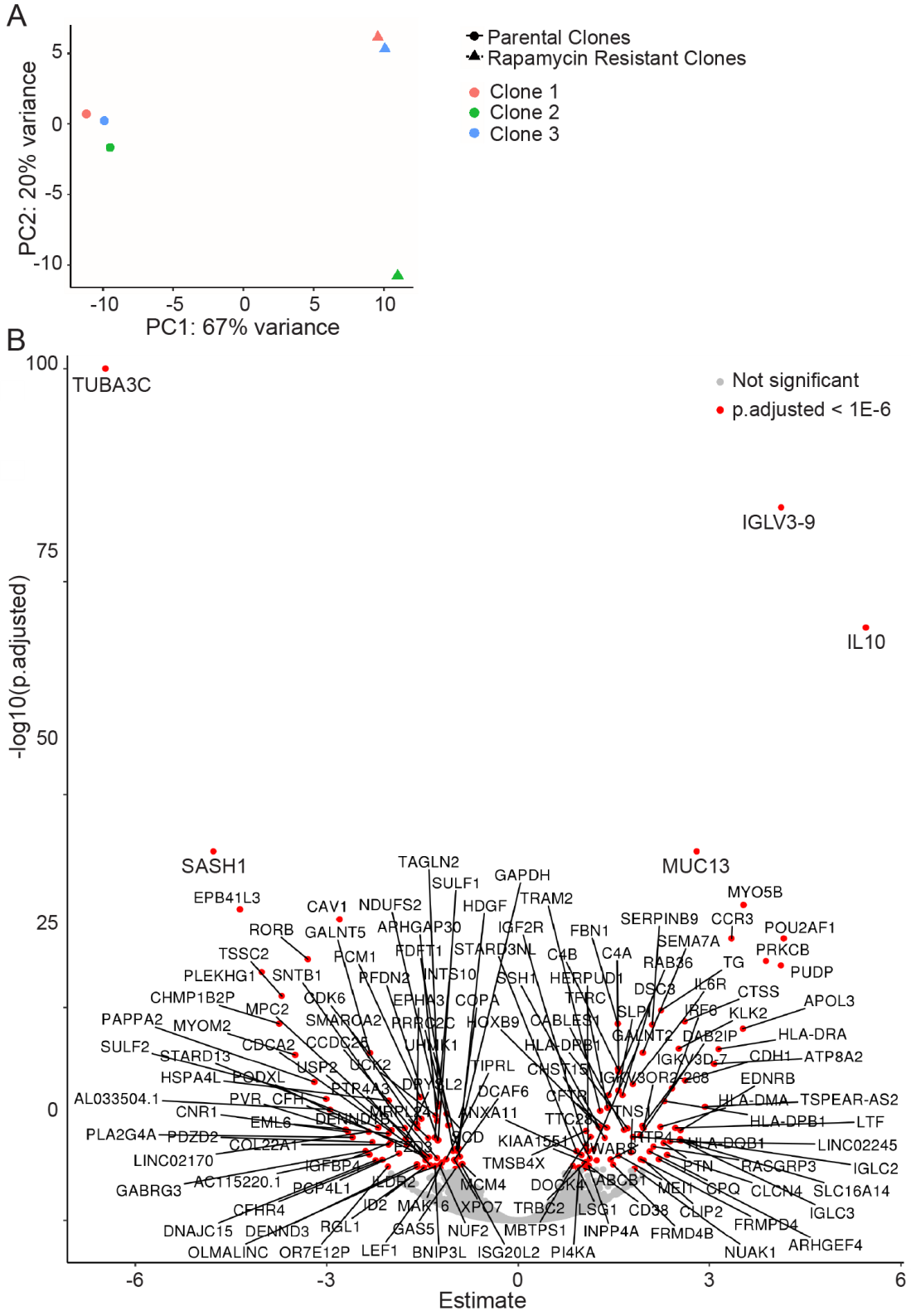
**

**Supplemental Figure 7:** (A) Principal component analysis (PCA) of rapamycin resistant clones (n=3) displayed as triangles and parental clones (=3) displayed as circles. Total 2-dimensional PCA mapping represent 87% of variance (PC1 = 67% and PC2 = 20%). (B) Volcano plot displaying differential expressed genes between rapamycin resistant and parental clones. The y-axis corresponds to the mean expression value of log 10 (p-value), and the x-axis displays the log_2_ fold change value. The red dots represent the most significant (p < 1x10^-6^) transcripts with differential expression between rapamycin resistant and parental clones. The grey dots represent the non-significant transcripts (p > 1x10^-6^). between rapamycin resistant and parental clones.
